# p-ANCA-Associated Vasculitis Caused by Levamisole-Adulterated Cocaine: A Case Report

**DOI:** 10.1155/2013/878903

**Published:** 2013-02-07

**Authors:** Michelle R. Carter, Sorour Amirhaeri

**Affiliations:** Department of Emergency Medicine, Howard University Hospital, 2041 Georgia Avenue, Washington, DC 20060, USA

## Abstract

A rare case of a patient with an unusual skin rash after using cocaine is presented. A clinical diagnosis of levamisole-induced vasculitis was made based on initial presentation of purpuric skin lesions involving the ears and positive cocaine on urine toxicology screening. The diagnosis was confirmed after laboratory findings of neutropenia, positive serum levamisole, and a histopathologic test of the skin lesions. The pathophysiology of this condition is discussed. Treatment with methylprednisolone and prednisone led to the resolution of the neutropenia and complete recovery of the skin lesions. With the growing use of levamisole-tainted cocaine, clinicians should be aware of the symptoms of vasculitis and neutropenia induced by this combination of drugs to avoid unnecessary tests and delayed diagnosis.

## 1. Background

Levamisole is an anthelmintic medication that was originally used to treat worm infestation in human and animals. It also has immunomodulatory properties and was used in the treatment of colon and breast cancers, rheumatoid arthritis, and nephrotic syndrome [1, 2]. Levamisole suppresses white blood cell production and leads to neutropenia, agranulocytosis, and thrombotic vasculopathy which results in retiform purpura first observed in children treated with this drug for nephrotic syndrome [1]. Levamisole is now banned by the FDA from use in humans in the United States. It is currently used mainly by veterinarians as an antiparasitic agent in animals.

Levamisole has also been used illegally in recent years as a cutting agent in cocaine to enhance its euphoric properties among users. It is a white powder that easily blends with cocaine but is not simply filler as it acts on the same receptors. It has been conjectured that it prolongs the effect of cocaine by increasing dopamine in euphoric centers of the brain [3]. There have been reports that vasculitis and retiform purpura induced by levamisole-tainted cocaine [4, 5] are also associated with the presence of circulating autoantibodies [1, 6].

In this report, we describe the diagnosis and treatment of a severe vasculitis in a patient who smoked levamisole-adulterated cocaine, and we suggest a mechanism to explain this association.

## 2. Case Presentation

A 43-year-old African American female presented to our Emergency Department (ED) with multiple painful purpuric skin rashes on her face, ears, and extremities that started 3 days before. The skin lesions started on the legs and later progressed to the thighs, arms, face, and ears and became confluent with some areas beginning to slough. The patient had associated odynophagia with mild dysphagia. She stated that she had a similar episode of a lesser degree a few months earlier but did not seek treatment. She was recently seen at a different hospital for an infected skin ulcer of her right knee and was treated with trimethoprim and sulfamethoxazole. The patient denied fever, chills, chest pain, and shortness of breath, cough, headache, blurred vision, or neck stiffness. Her only complaint was the painful lesions, and she asked for pain medication. She was homeless, and she reported a history of smoking crack cocaine for the past 20 years and had used it as recently as a day before admission to our ED. Her past medical history was significant for hypertension. She denied any medication use and had no known allergies.

**Figure 1:**
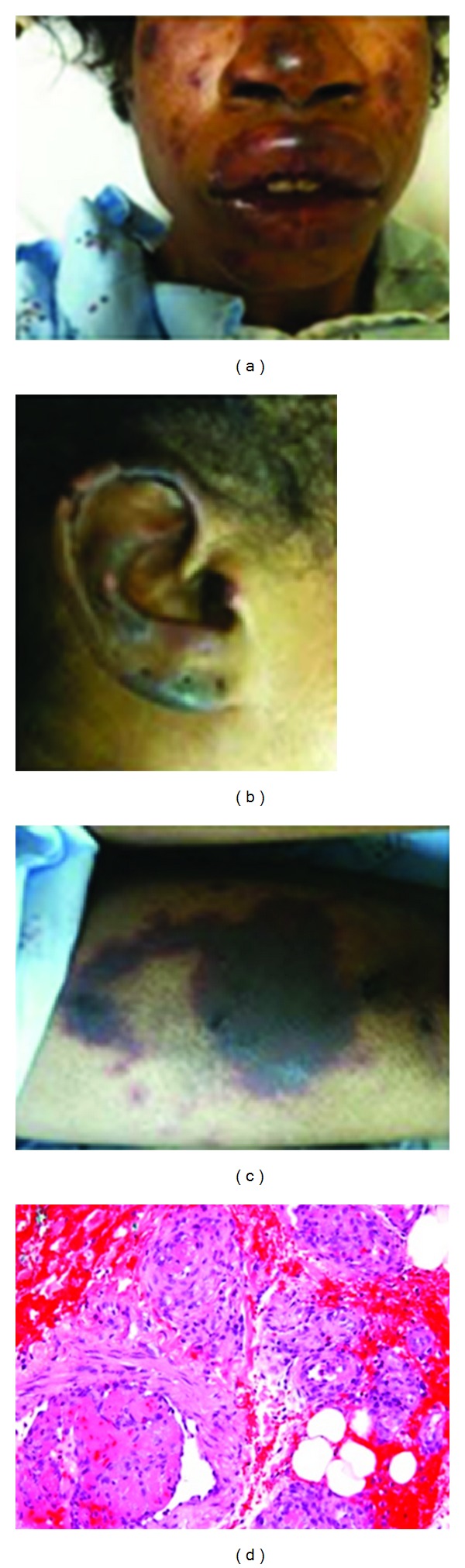
Skin lesions of the (a) face, (b) right ear, and (c) left thigh and (d) skin biopsy of the left thigh showing fibrin thrombin occluding the small-sized vessels with the presence of neutrophils and lymphocytes within the vessel wall.

On physical examination, she was an afebrile, cachexic female, in no acute distress. Her vital signs were blood pressure of 195/102 mmHg, pulse of 94 beats per minute, respiratory rate of 20 breaths per minute, temperature of 97.8° F, and oxygen saturation of 100% on room air. Cardiovascular, pulmonary, and abdominal examinations were unremarkable. Ear, nose, and throat examination revealed hyperemic purpuric lesions on her face, ears, and malar area with swollen lips. Dermatological examination revealed multiple confluent purpuric reticular plaques on her thighs, face, and ears with characteristic patterns containing central necrosis and erythematous borders (Figures 1(a), 1(b), and 1(c)).

Initial laboratory studies revealed neutropenia (WBC of 1,900 cells/mL, with 42% neutrophils and an absolute neutrophil count of 800.00) and increased sedimentation rate, with normal chemistry and coagulation panels. Toxicology screening of the urine was positive for cocaine. Urine revealed gross hematuria, and full analysis showed hematuria with numerous red blood cells and negative urine culture. Theserum levamisole level, detected by liquid chromatography tandem mass spectrometry, was positive. Treatment in the ED consisted of a normal saline bolus and 2 mg intravenous (IV) morphine sulphate for pain control, followed by 125 mg IV methylprednisolone, 25 mg IV antihistamine, and 50 mg IV ranitidine.

The dermatology service was consulted, and a presumptive diagnosis of levamisole-induced vasculitis was made based on the initial presentation of the lesions involving the ears and positive cocaine metabolites on urine toxicology screening. The patient was admitted to our hospital and started on 25 mg IV antihistamine every 6 hrs as needed for pruritus, 40 mg daily oral pantoprazole, and 125 mg IV methylprednisolone every 4 hrs for 2 days, followed by 80 mg every 8 hrs for 2 more days and finally a tapering daily oral dose of 30 mg prednisone. A biopsy specimen of purpuric skin lesions on her left thigh revealed fibrin thrombi in small-sized vessels consistent with vasculitis (Figure 1(d)). Laboratory results showed the following: HIV antibody negative, normal lymphocyte subset panel, RPR nonreactive, hepatitis B surface antibody negative, anticardiolipin antibody negative, ANA negative, RF negative, and HLA-B27 negative. The perinuclear pattern of ANCA (p-ANCA) was detected, consistent with ANCA-positive vasculopathy. These clinical findings also suggest toxic effects of levamisole.

After one week of hospitalization, the patient's WBC count normalized, and her skin lesions improved significantly. She was discharged with a tapering dose of prednisone and counseled to cease cocaine abuse. Arrangements were made for treatment at a rehabilitation unit. The patient was instructed to have a followup in the medicine and dermatology clinics of our hospital in 1-2 weeks.

The patient returned to our ED a few months later complaining of a recurrent painful skin rash on her nose and stated that the symptoms began 3 days prior after smoking crack cocaine, which she said was “cut with lamusol”. Laboratory results again showed neutropenia, reinforcing our original diagnosis of levamisole toxicity. Her WBC count improved with IV methylprednisolone, and she was discharged 3 days later with tapering prednisone. After counseling about cocaine use, she was treated at a rehabilitation unit.

## 3. Discussion

It is estimated that nearly 2 million people use cocaine in the United States [7]. Levamisole adulterated cocaine was first reported in 2002, and since that time, the percentage of cocaine contaminated by levamisole in Europe and the United States has risen steadily to about 82% [7].

This case describes a patient with vasculitis and skin rash associated with the use of levamisole-tainted cocaine concluded from positive urine analysis for cocaine and tandem mass spectrometry and gas chromatography for the presence of levamisole. The same symptoms occurred in the same patient with a repeat exposure to cocaine that she said was mixed with levamisole. The association of vasculitis with levamisole has been reported previously in patients who were treated with the drug for rheumatoid arthritis [8] and breast cancer [9] and in children with nephrotic syndrome [1]. In all cases, the lesions disappeared, and the WBC returned to normal values once the drug was discontinued.

It is suspected that levamisole affects the neurotransmission of the brain and enhances its euphoric and addictive properties by increasing dopamine [4]. Due to its short half life of about 5.6 hrs, it is often undetected or difficult to find after about 48 hours since it was ingested, except with very sensitive techniques such as tandem mass spectroscopy or gas chromatography which are often unavailable. A negative test does not rule out its presence or its causative role in recognizing the symptoms and final diagnosis. One product that levamisole decomposes into is 6-phenyl-2,3-dihydroimidazole 2, 1 thiazole, which is responsible for its lymphocyte-stimulating effects and has been found in samples analyzed by the FDA [7]. However, in a clinical setting, the presence of levamisole in adulterated cocaine is usually inferred by symptoms of vasculitis and retiform purpura that surface days after it was ingested. Skin lesions can appear anywhere in the body but preferentially in the ear, nose, and face with necrotic regions that give the appearance of a flesh-eating disease.

Biopsy of purpuric lesions in patients usually reveals a pattern of thrombotic and/or leukocytoclastic vasculitis. Retiform purpura has also been associated with the presence of circulating autoantibodies [1, 6] including p-ANCA in users of levamisole-tainted cocaine [6, 10, 11]. In one study, p-ANCA was detected in four of five consecutive cases of cutaneous vasculitis induced by levamisole-adulterated cocaine [10].

In our patient, the underlying pathophysiology of retiform purpura is related to thrombotic vasculitis detected from the histopathology of the skin lesions together with the presence of p-ANCA in the blood sample. We suggest that the retiform purpuric lesions were an autoimmune reaction to the ingestion of levamisole-tainted cocaine. We further suggest that levamisole triggers the production of p-ANCA, which binds to and degranulates neutrophils, producing chemotoxins that injure blood vessels resulting in vasculitis. This leads to superficial necrosis in areas that have a more tenuous blood supply, causing blockage of the capillaries, skin necrosis, and purpuric lesions.

This case highlights the serious consequences of the growing use of cocaine adulterated with levamisole. The fact that nearly 80% of the cocaine on sale in the US is contaminated with levamisole means that the problem is no longer confined to a few areas where cocaine is used. With the rise in cocaine addiction worldwide, clinicians should be aware of adulterated cocaine as a public health problem and be able to recognize that characteristic retiform purpuric skin lesions involving ears, face, and extremities in cocaine users may represent vasculitis induced by levamisole-tainted cocaine. Early recognition and treatment with methylprednisolone and prednisone results in complete resolution of skin lesions.

## References

[1] Rongioletti F, Ghio L, Ginevri F (1999). Purpura of the ears: a distinctive vasculopathy with circulating autoantibodies complicating long-term treatment with levamisole in children. *British Journal of Dermatology*.

[2] Mutch RS, Hudson PR (1991). Levamisole in the adjuvant treatment of colon cancer. *Clinical Pharmacology*.

[3] Buchanan JA, Oyer RJ, Patel NR (2010). A confirmed case of agranulocytosis after use of cocaine contaminated with levamisole. *Journal of Medical Toxicology*.

[4] Chung C, Tumeh PC, Birnbaum R (2011). Characteristic purpura of the ears, vasculitis, and neutropenia—a potential public health epidemic associated with levamisole-adulterated cocaine. *Journal of American Academy of Dermatology*.

[5] Lung D, Lynch K, Agrawal S (2011). Images in emergency medicine. *Annals of Emergency Medicine*.

[6] Laux-End R, Inaebnit D, Gerber HA, Bianchetti MG (1996). Vasculitis associated with levamisole and circulating autoantibodies. *Archives of Disease in Childhood*.

[7] Centers for Disease Control and Prevention (CDC) (2009). Agranulocytosis associated with cocaine use-four states. *MMWR Morbidity and Mortality Weekly Report*.

[8] MacFarlane DG, Bacon PA (1978). Levamisole-induced vasculitis die to circulating immune complexes. *British Journal of Medicine*.

[9] Scheinberg MA, Bezerra JBG, Almeida FA, Silveria LA (1978). Cutaneous necrotizing vasculitis induced by levamisole. *British Medical Journal*.

[10] Ullrich K, Koval R, Koval E (2011). Five consecutive cases of a cutaneous vasculopathy in users of levamisole adulterated cocaine. *Journal of Clinical Rheumatology*.

[11] Mansi IA, Opran A, Rosner F (2002). ANCA-associated small-vessel vasculitis. *American Family Physician*.

